# Diet composition of the African manatee: Spatial and temporal variation within the Sanaga River Watershed, Cameroon

**DOI:** 10.1002/ece3.8254

**Published:** 2021-11-02

**Authors:** Aristide Takoukam Kamla, Dylan G. E. Gomes, Cathy A. Beck, Lucy W. Keith‐Diagne, Margaret E. Hunter, Ruth Francis‐Floyd, Robert K. Bonde

**Affiliations:** ^1^ African Marine Mammal Conservation Organization Edea Cameroon; ^2^ Department of Large Animal Clinical Sciences College of Veterinary Medicine University of Florida Gainesville Florida USA; ^3^ Department of Biological Sciences Boise State University Boise Idaho USA; ^4^ U.S. Geological Survey Wetland and Aquatic Research Center Gainesville Florida USA; ^5^ African Aquatic Conservation Fund Joal Senegal; ^6^ Clearwater Marine Aquarium Research Institute Clearwater Florida USA; ^7^ Present address: Cooperative Institute for Marine Resources Studies Hatfield Marine Science Center Oregon State University Newport Oregon USA

**Keywords:** endangered species, marine mammal, protection, Sirenia, threatened, Trichechidae, Trichechus senegalensis, vulnerable

## Abstract

The present study aimed to investigate the diet of African manatees in Cameroon to better inform conservation decisions within protected areas. A large knowledge gap on diet and seasonal changes in forage availability limits the ability to develop informed local management plans for the African manatee in much of its range. This research took place in the Sanaga River Watershed, which includes two protected areas in the Littoral Region of Cameroon: the Douala‐Edea National Park and the Lake Ossa Wildlife Reserve. We analyzed 113 manatee fecal samples and surveyed shoreline emergent and submerged vegetation within the Sanaga River Watershed. We used microhistological analyses to determine the relative contribution of each plant species to African manatee diets and compared across locations and across seasons (wet vs. dry season). We found that the shoreline vegetation is diverse with over 160 plant species, unevenly distributed across space and season, and dominated by emergent vegetation mostly represented by the antelope grass (*Echinochloa pyramidalis*). We recorded a total of 36 plant species from fecal samples with a spatial and temporal distribution mostly reflecting that of the corresponding shoreline vegetation. African manatees appear to be primarily opportunistically feeding on available vegetation across the seasons and habitat. This work documents the current, but changing, state of plant availability in the Sanaga River Watershed and reports the African manatee diet in Cameroon for the first time. This information can play a critical role in successfully managing the species and these protected areas. If we wish to protect the African manatee and the aquatic ecosystems within the Sanaga River Watershed, we must understand how forage availability changes over time, especially as its waters become nutrient enriched, eutrophic, and exposed to invasive species of plants in a changing world.

## Graphical abstract

Due to a large knowledge gap on diet and seasonal changes in forage availability, this study aims to investigate the diet of threatened African manatees to better inform conservation decisions within protected areas of Cameroon. We analyzed 113 manatee fecal samples and surveyed shoreline emergent and submerged vegetation within the Sanaga River watershed and found that African manatees appear to be primarily opportunistically feeding on available vegetation across the seasons and habitat. If we wish to protect the African manatee and the aquatic ecosystems within the Sanaga River watershed, we must understand how forage availability changes over time, especially as its waters become nutrient enriched, eutrophic, and exposed to invasive species of plants in a changing world.

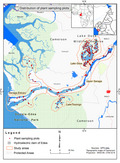

## 1 INTRODUCTION

The Order Sirenia, also known as sea cows, consists of four extant aquatic and hindgut fermenter herbivorous species: the West Indian manatee, the Amazonian manatee, the dugong, and the African manatee. The African manatee is the least studied of all sirenians (Marsh et al., 2011). They are threatened by poaching, accidental catches in fishing nets, entrapment by dams, and habitat degradation—despite legal protection in all 21 countries within their range, as well as international laws (Powell, 1996). They are Red‐listed by the International Union for Conservation of Nature (IUCN) as “Vulnerable” and belong to Appendix I of the Convention on International Trade in Endangered Species (CITES) and of the Convention on Migratory Species (CMS).

Understanding the influences on a species' distribution and its seasonal diet can be crucial for effectively managing wildlife populations. The availability of food resources is an essential component of a species' ecological niche and contributes to the species' distribution patterns (Cox & Moore, 2000). A large knowledge gap on diet and seasonal changes in forage availability limits the ability to develop informed local management plans for the African manatee in much of its range. Plant species composition and feeding strategies of the African manatee differ widely across habitats (Keith‐Diagne, 2014; Powell, 1996); thus, management plans must consider local food availability and dietary needs of local manatee populations.

Akoi (2004) examined 35 African manatee fecal samples from the Ivory Coast and corroborated earlier findings of Best (1983) that the African manatee diet is composed predominantly of grasses but also includes fruit and deposited organic material—particularly during the dry season when decreased water levels limit access to emergent vegetation. In later work, Keith‐Diagne (2014) determined the lifetime diet composition of 24 African manatees via carbon and nitrogen stable isotope analysis of ear bones from carcasses recovered in Senegal and Gabon. The stable isotope signatures recorded from samples collected in Gabon indicated that their diet was made up of 90–94% of plants and 6–7% of hermit crabs. The diets from Senegal, however, differed substantially and were composed of 46–57% of plants, 24–27% of fish, and 19–24% of mollusks. This omnivorous behavior, which has been anecdotally reported by local fishermen in many countries (Dodman et al., 2008; Mayaka et al., 2019; Powell, 1996; Reeves et al., 1988; Takoukam Kamla, 2012), had been previously considered opportunistic, only when plants are not available, but these results from Senegal suggest otherwise.

**FIGURE 1:**
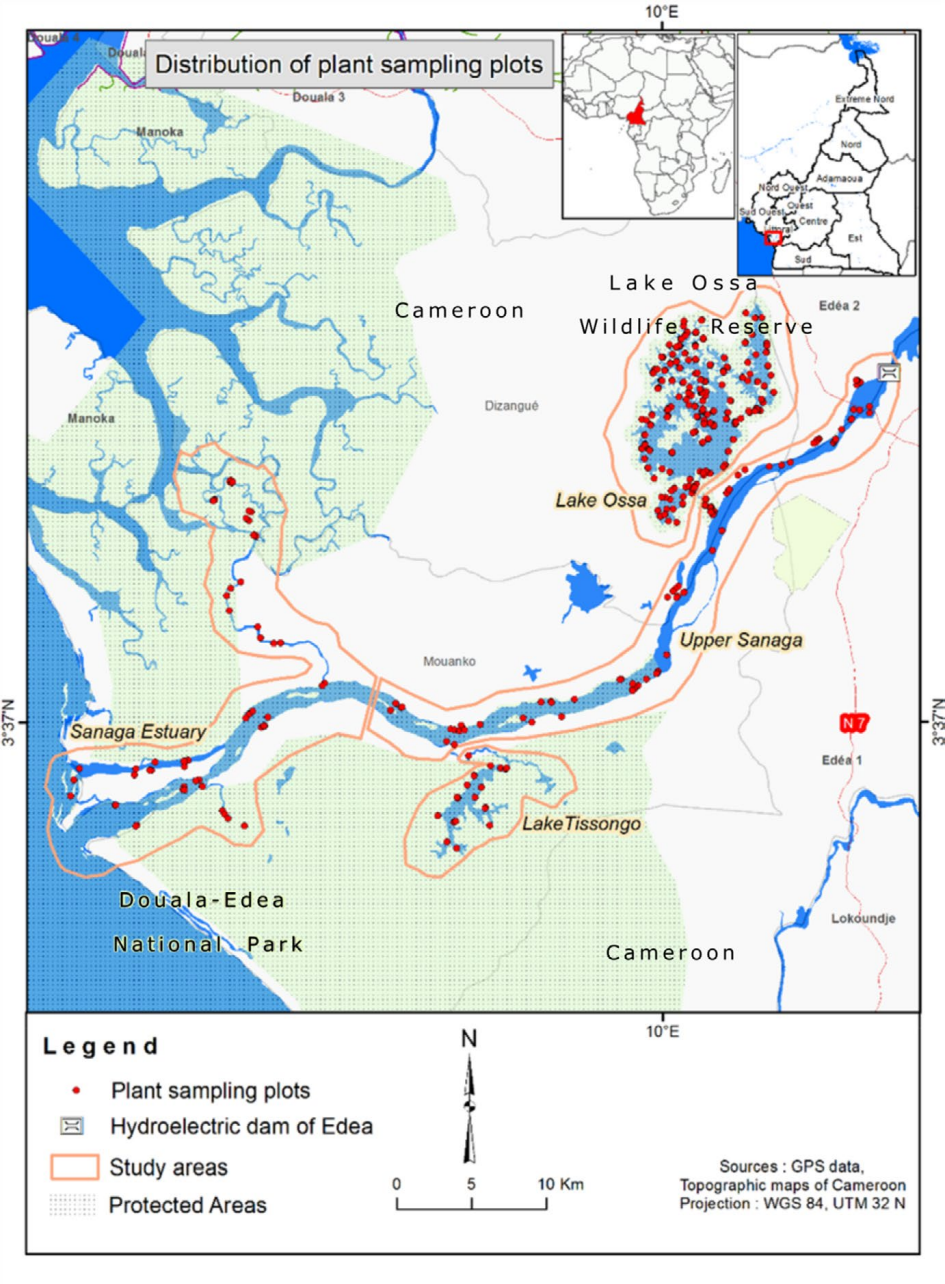
Map of the Sanaga River Watershed, downstream of the hydroelectric dam of Edea, showing the surveyed areas and the distribution of the surveyed plots

In Cameroon, manatees are present in the lower reaches of most rivers and lakes with a direct connection to the Atlantic Ocean. Lake Ossa and the reaches of the Sanaga River downstream of the hydroelectric dam of Edea provide a dry season sanctuary for manatees within the watershed (Powell, 1996) (Figure 1). These areas encompass two adjacent protected areas including the Lake Ossa Wildlife Reserve and the National Park of Douala‐Edea, respectively, which were set aside for manatee conservation. Yet, the African manatee is an elusive and cryptic species, living in the turbid waters of the Sanaga River Basin where water transparency in most places is less than a meter (Takoukam Kamla et al., 2021). Due to the poor visibility, it is difficult to conduct direct observational studies on the feeding behavior and diet of the African manatee in these areas. To establish an efficient management plan, information on manatee use of the habitat and diet composition is needed for the two protected areas. Results of this study aim to identify important manatee use areas within the protected areas, areas for habitat restoration, and to help inform conservation efforts to ensure the long‐term survival of manatees in this region.

The purpose of this study is to contribute to the conservation of the African manatee in the Sanaga River Watershed by determining manatee diet composition and characterizing the aquatic vegetation present in the area. More specifically, the goals of this study were to (a) document aquatic plants present in the Sanaga River Watershed, (b) document the diet composition of African manatees in the Sanaga River Watershed, and (c) assess differences and similarities in diet composition by locations or habitats and water levels (seasonal variation).

## 2 METHODS

### 2.1 Study area

The Sanaga River Watershed encompasses two protected areas in the Littoral Region of Cameroon (Figure 1). The Douala‐Edea National Park (3°14′ 3°50′N; 9°34′–10°03′E) was created in 1932 and covers approximately 160,000 ha of land and water (Blaikie & Simo, 2000). The park stretches along both sides of the lower reaches of the Sanaga River and 100 km along the Atlantic coastline of Cameroon (Feka et al., 2009). The park surface is predominantly a tropical lowland equatorial forest (80%) and covered by about 6.4% of mangrove, which is seriously threatened by deforestation. The local fishing community utilizes mangrove wood to smoke fish, which is the major economic activity in the area. The Lake Ossa Wildlife Reserve (3°45′–3°52′N; 9°45′–10°4′E) is a complex of lakes located about 13 km from Edea, Cameroon (Wirrmann & Elouga, 1998). The water surface is estimated to be 4000 ha, which represents about 90% of the Lake Ossa Wildlife Reserve. The reserve was established to provide a refuge for the protection of the African manatee.

### 2.2 Sampling design

We used boats to survey submerged and emergent vegetation along the shoreline of four areas within the Sanaga River Watershed (Figure 1) twice between June and September 2016 to characterize the vegetation. They were also frequently visited between June 2015 and November 2017 in search of free‐floating manatee fecal samples. These areas represented three habitat types: lake (Lakes Tissongo and Ossa), river (Sanaga River), and estuary (Sanaga Estuary; Figure 1). Plant surveys and manatee fecal collection were conducted during the high‐water season (average depth >2 m) in Lake Tissongo, the Sanaga River, and the Estuary, but were collected during both the high‐ and the low‐water seasons (average depth <2 m) in Lake Ossa. The low‐water season extends from November to May or June, and the high‐water season extends from July to October or November (Nguetsop et al., 2004).

### 2.3 Fecal sample collection

A total of 113 fecal samples (112 free‐floating manatee boluses and one hindgut sample taken from the lower intestine of a stranded calf carcass) were examined using microhistological analysis techniques, described below. The distribution of fecal collection sites in our study area is presented in Table 1 and Figure S1 in the Appendix S1. In Lake Ossa, 29 fecal samples were collected during the high‐water season and 31 during the low‐water season. Samples were preserved in 70% ethanol before examination (Allen et al., 2018; Hurst & Beck, 1988).

**TABLE 1:** Diversity index and related information of the plant species recorded in African manatee feces for each surveyed location within the Sanaga River Watershed

	Sanaga Estuary	Upper Sanaga	Lake Tissongo	Lake Ossa	All sites
Sample size	33	9	11	60	113
Minimum number of plant species	24	15	16	24	32
Species diversity	1.81	1.53	1.58	1.45	1.83
(Shannon index *H*)					

### 2.4 Habitat characterization and plant library collection

In order to understand the variety of plant species accessible to the African manatee in each location, the shoreline vegetation was surveyed, and representative samples were collected. A total of 238 100‐m transects were evenly spaced along the shoreline of the study area. Each transect consisted of two to six 1 × 1‐m plots for a total of 958 plots distributed across the four study locations (Table 2; Figure 1), and the plots were placed within two meters of the shoreline in areas that were considered accessible by manatees. Plant samples were collected from each plot and identified by an expert plant taxonomist. The dried plants were preserved in newspapers. Finally, the percent coverage by plants in the plot was estimated.

**TABLE 2:** Survey effort and diversity index of shoreline vegetation by location in the lower Sanaga River Watershed

	Sanaga Estuary	Upper Sanaga	Lake Tissongo	Lake Ossa	All sites
Number of plots surveyed	80	146	64	668	958
Number of species	68	96	16	111	160
Species diversity (Shannon index *H*)	3.09	3.55	1.86	2.61	3.52

#### 2.4.1 Reference Plant slide preparation

Reference slide preparation was done following the protocol described in Hurst and Beck (1988) with some modifications. The epidermis of the leaf and sometimes stems of frequently encountered plant species within the survey area were mounted on a microscope slide for identification under 100× and 400× magnification. The leaf or the stem was scraped carefully with a scalpel; then, Visikol® for Plant Biology™ solution was used to clear out the chlorophyll to improve the optical quality of the specimen. No staining was necessary as most of the microhistological characters were visible after clearing. Specimen material was permanently mounted on glass slides using a Visikol® MOUNT™ solution. The slides were dried for two to four days before microscopic examination.

#### 2.4.2 Photography

A 14 Megapixel OMAX (Model: A35140U) microscope USB digital camera was used to take photomicrographs of plant material. The digital camera was mounted on the microscope through an OMAX fixed microscope adapter (Model: A3RDF50) with a 0.5× reduction lens that increased the field of view. Photomicrographs were captured using both 10× and 40× objective magnifications and under standard bright field illumination as well as polarized light to reveal glowing microhistological characters. A digital catalog of the photomicrographs was organized by plant family, and priority was given to the most abundant plant species and plants suspected of being manatee food.

### 2.5 Microhistological analysis

Fecal samples were examined using microhistological features of undigested plant fragments and were analyzed using the techniques described by Hurst and Beck (1988). The digested samples were washed free of dirt and fine particles with tap water over a 30 mesh (0.52 mm) screen. A small quantity (about 100 mg) of washed sample was spread uniformly over a 2 × 3 inch slide, to which drops of Hertwig's solution or Visikol® for Plant Biology™ optical clearing solution were added. When necessary, the slide was heated over an alcohol flame to clear pigments from the plant cells, providing a better view of cellular structures. Five slide replicates were performed for each fecal sample. The slides were observed under a microscope at 100× magnification. Plant fragments were identified by comparing their histological structures with voucher microscope slides, photomicrographs, or illustrations from references. For fragments that could not be identified but with distinctive microscopic features, a generic name was assigned, for example, “Unidentified 1.” The same generic name was given to fragments with the same features.

### 2.6 Quantification of percent food plant species occurrence

Each of the five slides for each fecal sample was examined using 20 different fields of view indicated by the vertical and horizontal graduations of the mechanical stage as described by Hurst and Beck (1988). Each of the fields of view was observed within a gridded frame of the microscope eyepiece whereby vertical lines represent randomly numbered (1–11) transect lines. Transect line 1 is at the center of the grid, with even transect lines on the left and odd lines on the right. Plant fragments at the interception of a transect line with the horizontal lines were recorded starting from transect line 1. Intercepts on a transect line were examined subsequently until five plant fragment records were achieved. When the latter was not achieved on transect line 1, the observer continued to the next transect line number.

The occurrence of each plant species on a slide was counted, then the percent occurrence in a fecal sample was obtained by tallying occurrence for each species from each of the five slides. Thus, each fecal sample was examined at 500 intercept points (5 slides × 20 fields of view × 5 intercepts). The percent occurrence of each food plant species was averaged by location, season, and a categorical feces size (i.e., small, medium, large).

### 2.7 Data analysis

#### 2.7.1 Habitat shoreline species characterization

All data were analyzed using EXCEL Data Analysis tools and XLSTAT‐Ecology (De Levie, 2004; Guerrero, 2019; Middleton, 1996) and R (R Core Team, 2017). Plot locations were mapped using ArcGIS (version 10.6). For each location, the number of unique plant species encountered, and the relative abundance of each species, was computed by averaging the percent occurrence per plot by the total number of plots for that location. The relative abundance of the plant families and types was also computed. The species diversity of the shoreline vegetation was estimated using the Shannon diversity index *H* (Shannon, 1948).

(1) $$H=‐\sum_{i=1}^{n}P_{i}\ln\left(P_{i}\right)$$

where *n* is the total number of species, *P_i_* the proportion of each species, *i*.

The difference in species composition among locations was measured using the Bray–Curtis dissimilarity matrix (Bray & Curtis, 1957). In order to buffer the influence of strongly dominate species, the relative abundance of each species was standardized by first taking the logarithm of the relative abundance before calculating the distance matrix (Kindt & Coe, 2005).

The analysis of similarities (ANOSIM) was performed on the PAST 3.24 software (Hammer et al., 2001) to test for the significance of the difference in species composition between location and sites. ANOSIM is a non‐parametric statistical test similar to an ANOVA test which uses a permutation and randomization methods from a ranked similarity matrix to generate the R‐statistics that determines whether the similarity between groups is greater than or equal to the similarity within groups (Clarke, 1993). All ANOSIM tests in this study were performed with 10,000 permutations.

The similarity of percentages (SIMPER) computed the contribution of each species in the dissimilarities between locations and seasons using the Bray–Curtis similarity index of the most frequent species. The Bonferroni correction was applied to compensate for errors due to the multiple comparisons test (Clarke, 1993). Shoreline species composition and diversity were also compared between seasons for Lake Ossa as it was the only location surveyed during both the low‐ and high‐water seasons.

#### 2.7.2 Diet composition and diversity analysis

The same metrics (relative abundance, number of species, Shannon diversity index) used to describe the shoreline vegetation were also used to characterize and compare diet composition between locations, seasons (Lake Ossa only), and the feces bolus size. Feces were categorized as small or large depending on their diameter (≤4 cm, and >4 cm, respectively). The cutoff value was determined by building a frequency distribution histogram of the diameter of 377 fecal samples collected during this study. Each of the top five dominant identified plant food species was compared between locations, seasons, and fecal sizes using a Kruskal–Wallis test since the distribution of relative abundance failed the test for normality (Kruskal & Wallis, 1952).

A Venn diagram was built through web‐based software (Heberle et al., 2015) to analyze the inclusivity and exclusivity between diet and potential food plants by location.

## 3 RESULTS

### 3.1 Shoreline vegetation characterization

Among the 958 plots surveyed during this study, 160 plant species, 122 genera, and 43 families were recorded along the shorelines of the four locations (Lake Ossa, Lake Tissongo, Sanaga Estuary, and Upper Sanaga). The highest number of species was recorded in Lake Ossa (111), followed by Upper Sanaga (96), Sanaga Estuary (51), and Lake Tissongo (16) (Table 2). The plateau on the species accumulation curves for each location (Figure S2) indicates that our survey effort captured nearly the maximum number of species present in each location except in Lake Tissongo. The Shannon diversity index (*H*) varied across locations with the highest values recorded in Upper Sanaga (*H* = 3.55), Sanaga Estuary (*H* = 3.09), and the lowest value in Lake Tissongo (*H* = 1.86).

In the comparison of the surveyed shoreline plant species composition using the ANOSIM, there was a significant difference between locations (*R* = 0.35; *p *< .0001), indicating a strong effect of the habitat type on the plant species composition. The pairwise comparison of species composition by location indicated a significant difference between each location pair (*p *< .001 for each pair). The Bray–Curtis pairwise coefficients of dissimilarity also indicated a high difference in species composition and relative abundance between locations. The Bray–Curtis distance was greatest between Lake Ossa and Sanaga Estuary (87% of dissimilarity) and lowest between the Upper Sanaga and Sanaga Estuary (66% of dissimilarity; see Table 3). *A posteriori* comparisons using the SIMPER method allowed estimation of the relative contribution of each plant species to the observed significant differences between locations (indicated in Table 4). *Echinochloa pyramidalis* had the highest contribution (34.45%) in the difference of species composition between locations. The overall average dissimilarity computed by the SIMPER method between locations was 90.36%.

**TABLE 3:** Pairwise coefficient of dissimilarity (Bray–Curtis distance) of shoreline plant communities between four locations in the Sanaga River Watershed

	Sanaga Estuary	Upper Sanaga	Lake Tissongo	Lake Ossa
Sanaga estuary	0.00			
Upper Sanaga	0.66	0.00		
Lake Tissongo	0.82	0.76	0.00	
Lake Ossa	0.87	0.68	0.69	0.00

**TABLE 4:** Major plant species surveyed along the shoreline of the Sanaga River Watershed and their relative contribution to the Bray–Curtis similarity index between Sanaga Estuary, Upper Sanaga, Lake Tissongo, and Lake Ossa

Species	Average abundance %				Contribution %	Cumulative %
	Sanaga Estuary	Upper Sanaga	Lake Tissongo	Lake Ossa		
*Echinochloa pyramidalis*	3.25	15.8	16.8	46	34.45	34.45
*Dissotis erecta*	2.44	1.89	27.1	5.15	9.331	43.78
*Eremospatha macrocarpa*	0.625	0	32.3	2.07	7.155	50.93
*Alchornea cordifolia*	10.6	4.32	6.72	0.531	5.626	56.56
*Ipomoea alba*	0	9.35	0	1.59	4.792	61.35
*Rhizophora racemosa*	19.9	0	0	0	4.562	65.91
*Pennisetum purpureum*	5.81	6.61	0	0.322	4.177	70.09
*Dissotis falcipila*	0	0.137	0	5.57	3.926	74.01
*Ficus capreifolia*	3.25	5.6	0	0.132	3.258	77.27
*Eichhornia crassipes*	8.31	3.8	0	0	3.105	80.38
*Ficus capreifolia*	0	5.14	0	1.03	2.83	83.21
*Canthium ciliatum*	0	0.685	0	3.82	2.813	86.02
*Ludwigia stolonifera*	0	0	0	3.24	2.553	88.57
*Laccosperma secundiflorum*	0	0	5.63	1.89	2.213	90.79
*Acroceras zizanioides*	1.25	1.71	0	1.13	1.837	92.62
*Polygonum lanigerum*	0.375	2.33	0	0.892	1.827	94.45
*Salvinia molesta*	0	0	0	2.15	1.564	96.01
*Fuirena umbellata*	0	0	1.56	1.89	1.535	97.55
*Laccosperma robustum*	0	0	0	2	1.287	98.84
*Cyperus haspan*	0.5	0	0.781	1.29	1.164	100
Overall average dissimilarity = 90.36%						

Note

Values were calculated using SIMPER (see methods).

**FIGURE 2:**
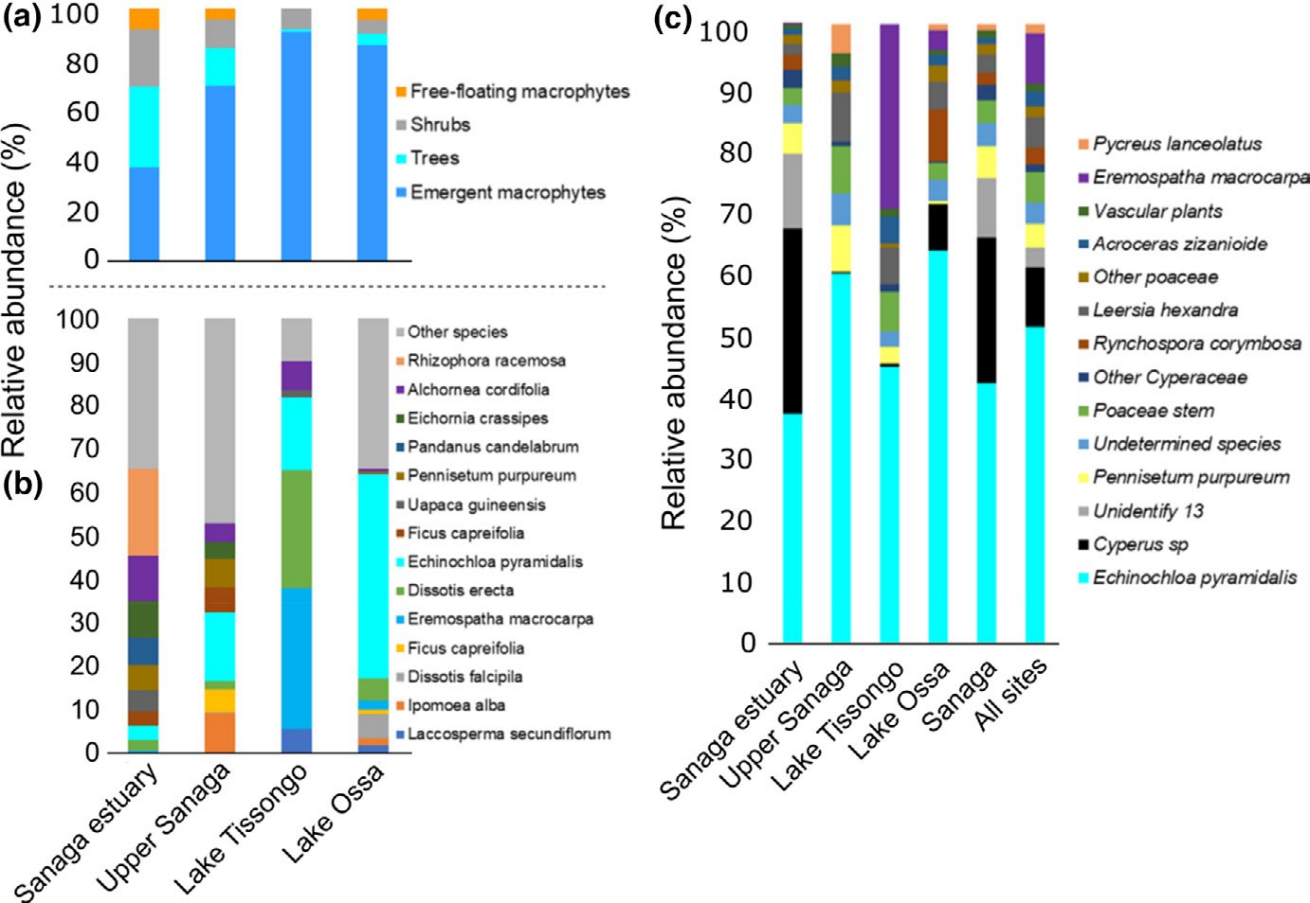
Vegetation composition profiles by location. (a) Shoreline vegetation percent by plant type across the four surveyed locations of the Sanaga River Watershed. (b) Shoreline plant species composition profile across the same surveyed locations as in panel (a). (c) Identified plant species composition from African manatee feces collected from the locations within the Sanaga River Watershed

Overall, the shoreline surveys identified emergent macrophytes as the highly dominant plant type (70.7%), then trees (13.0%), shrubs (11.9%), and the less abundant plant types—which were free‐floating macrophytes (4.3%; Table S1; Figure 2a), there was no submerged aquatic vegetation. The most abundant plant species across locations were *E. pyramidalis* (20.7%), *Dissotis erecta* (9.2%), and *Eremospatha macrocarpa* (8.8%) (Table 5). The relative abundance of emergent macrophytes was higher in Lake Tissongo (90.6%) and Lake Ossa (85.5%) and was lower in Upper Sanaga (69.5%) and Sanaga Estuary (37.4%). The Sanaga Estuary shoreline has the highest abundance of shrubs (22.6%), trees (31.7%) and free‐floating macrophytes (8.3%). The most abundant plant families along shorelines were Poaceae (27.5%), Arecaceae (12.0%), Euphorbiaceae (10.6%), Melastomataceae (10.7%), and Rhizophoraceae (6.3%) (Table 5). The abundance of Poaceae along shorelines varied by location and was highest in Lake Ossa where it represented 50.5% of the total species (Upper Sanaga = 30.2%; Lake Tissongo = 18.7%; Sanaga Estuary = 10.6%). The Lake Tissongo shoreline was highly dominated by Arecaceae (38.0%) and Euphorbiaceae (27.1%), while the Sanaga Estuary shoreline was dominated by Rhizophoraceae (19.9%) and Euphorbiaceae (11.6%) (Table S1).

**TABLE 5:** List of plant type, family, and species (with the common names) and their percent frequency in the 113 African manatee feces collected in the Sanaga River Watershed

Plant types	Common English names	*n*	%
Grasses			96.11
Poaceae	Grass		71.08
*Echinochloa pyramidalis*	Antelope grass	98	53.51
*Leersia hexandra*	Southern cutgrass, swim rice grass	32	4.08
Unidentified 13		8	3.53
*Poaceae* stem		43	3.48
*Pennisetum purpureum*	Elephant grass	28	2.62
*Acroceras zizanioides*	Oat grass	17	1.88
*Cynodon* sp.^a^	Bermuda grass, devil grass	8	1.23
*Hyparrhenia* sp.^a^	Jaragua grass, Giant thatching grass	12	0.52
*Panicum maximum*	Guinea grass	4	0.13
Unidentified 6		1	0.07
Unidentified 15		1	0.03
*Leptochloa* sp.	Sprangletops	1	0.01
Cyperaceae	Flatsedge		20.08
*Cyperus* sp.	Nut sedge, nut grass	26	12.85
*Rynchospora corymbosa*	Matamat	38	5.15
*Pycreus lanceolatus* ^a^	Epiphytic flatsedge	9	0.87
*Remirea maritima* ^a^	Beachstar	3	0.43
*Ascolepis* sp.^a^		5	0.34
*Other Cyperaceae*		10	0.21
Unidentified 2		3	0.09
Unidentified 8		2	0.08
Unidentified 4		2	0.06
Arecaceae	Palm		4.72
*Eremospatha macrocarpa* ^a^	Small rattan	31	4.72
Pontederiaceae	Pickerelweed		0.08
*Eichhornia crassipes*	Water‐hyacinth	2	0.08
Vascular plants			0.80
Asteraceae	Aster, Daisy, Sunflower		0.39
Acanthospermum sp.^a^	Starburr	7	0.22
Other Asteraceae		2	0.17
Fabaceae	Legume, pea, bean		0.24
*Centrosema pubescens* ^a^	Butterfly pea	4	0.17
*Dalbergia* sp.	Rosewood, Bombay, black wood	1	0.05
*Millettia mannii* ^a^	Ndu Ezi (Nigeria, Igbo)	1	0.02
*Albizia* sp.^a^	Silk tree	1	0.00
Lamiaceae	pure, deadnettle, sage		0.02
*Platostoma* sp.^a^	Chinese mesona	1	0.02
Nymphaeaceae	Water lilies		0.12
*Nymphaea lotus*	White Egyptian lotus, tiger lotus	1	0.12
Salviniaceae	Watermoss		0.03
*Salvinia molesta*	Giant Salvinia, Kariba weed, water fern	1	0.03
Other plants species			3.24
Fruits^b^			
*Eremospatha macrocarpa* ^c^	Small rattan		
*Calophyllum inophyllum* ^a^	Beach touriga		
*Canthium ciliatum* ^a^	Hairy turkey berry		
*Ficus copreifolia* ^a^	Fig shrub		
*Ficus rubiginosa* ^a^	Rusty fig		
*Macaranga* sp.	Parasol leaf tree		
Clay		1	0.71

Note

Lightly shaded rows represent plant Families, whereas darkly shaded rows represent larger taxonomic groups. *n* = the number of fecal samples that contained the focal plant.

^a^ Newly reported African manatee food plants.

^b^ Fruit pieces in feces were not observed using the microhistological analysis protocol described in the methodology. Each fecal sample (including those that were not selected for microhistological analysis in this study) was systematically visually examined to identify fruits, scales, and other debris present. Thus, we did not provide percent frequency and *n* value for the fruits identified.

^c^ Plant species already listed once above within Arecaceae.

The dominant plant species in Lake Ossa and the Upper Sanaga was *E. pyramidalis*, while the dominant plant species in Lake Tissongo and the Sanaga Estuary were *E. macrocarpa* and *Rhizophora racemosa*, respectively (Figure 2b). The Venn diagram in Figure S3 shows the number of shared and unique plant genera between locations. Five genera were found ubiquitous to all locations: *Echinochloa*, *Dissotis*, *Commelina*, *Uapaca*, and *Commelina*. Lake Ossa had the highest number of unique plant genera (26). Lake Ossa and Upper Sanaga had the highest number of shared genera (50).

**FIGURE 3:**
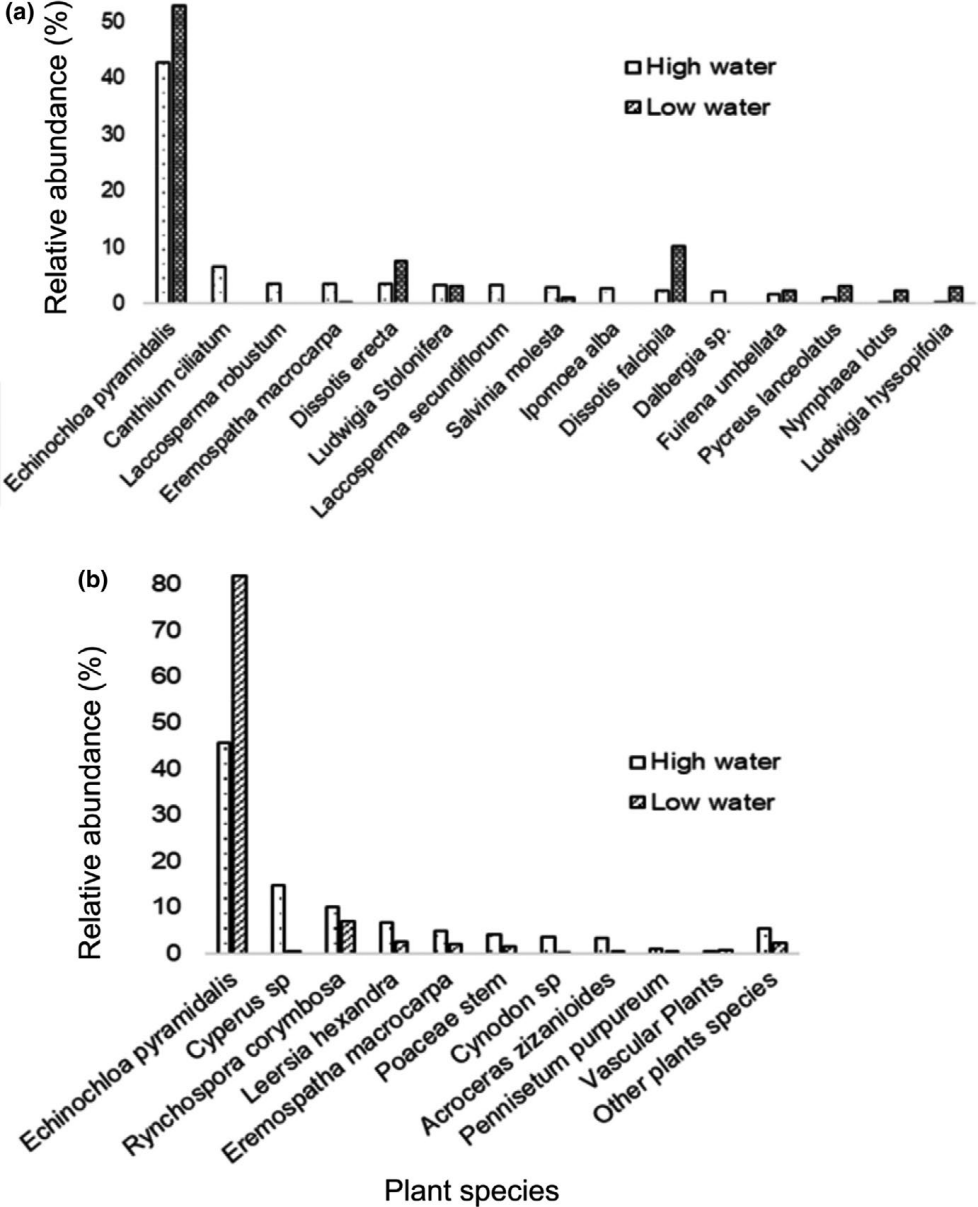
Relative abundance of top 15 plant species identified along the shorelines of Lake Ossa (a) and within African manatee feces (b) by season (water level)

Although there was no significant difference in shoreline plant species composition between seasons (based on the ANOSIM test) in Lake Ossa (*R* = −0.009; *p* = .86), the number of shoreline plant species encountered during the high‐water season (101) was 2.6 times higher than in the low‐water season (39). *Echinochloa pyramidalis*, *D. erecta*, and *D. falcipila* were more abundant during the low‐water than during the high‐water season (Figure 3a). The Shannon diversity index was higher (*H* = 2.74) during the high‐water than during the low‐water (*H* = 2.01) season.

### 3.2 Diet composition from African manatee fecal samples in the Sanaga River watershed

The number of unique plant species recorded in each fecal sample ranged from one to nine, with an average of four. Most plant fragments (89.2%) were identified to at least the genus level. Among the 113 fecal samples analyzed, vegetative material from 31 plant species (from nine plant families) consumed by the African manatee were observed (Table 5), in addition to fruits from six species and one emergent macrophyte. The greatest number of forage plant species was found in Lake Ossa (24) and the Sanaga Estuary (24), and the lowest number was found in the Upper Sanaga (15) and Lake Tissongo (16).

The most represented plant type was emergent grasses, which were present in all fecal samples and constituted 96.1% of the samples. Vascular plants constituted only 0.8% of samples. Three free‐floating plant species (*Nymphaea lotus*, *Salvinia molesta*, and *Eichhornia crassipes*) were found but were poorly represented (0.23%, all three species combined) and were present in only one fecal sample (Table 5). No submerged aquatic plants were found.

The three most abundant families were Poaceae (representing 71.1% of the overall diet), Cyperaceae (20.1%), and Arecaceae (4.8%). The five most abundant species were *E. pyramidalis* representing 53.5% of the fecal fragments, followed by *Cyperus* sp. (12.9%), *Rynchospora corymbosa* (5.15%), *E. macrocarpa* (4.7%), and *Leersia hexandra* (4.08%) (Figures 2c and 3b). These species were also the most frequent and occurred in 98 (80.5%), 26 (23.0%), 38 (33.6%), 31 (27.4%), and 32 (28.3%) of the 113 fecal samples, respectively (Table 4).

Among the 36 plants recorded from the 113 fecal samples analyzed in this study, 19, 9, and 8 were identified at the level of the species, genus, and family taxa, respectively. Among these plants, 14 genera were previously reported or documented in the list of African manatee food resources by (Keith‐Diagne, 2014). Those previously reported species include *E. pyramidalis*, *Acroceras zizanioides*, *Cyperus papyrus*, *Eichhornia crassipes*, *Dalbergia* sp., *L. hexandra*, *Nymphaea lotus*, *Pennisetum purpureum*, *Rhynchospora corymbosa*, *S. molesta*, *Leptochla* sp., *Panicum* sp., *Ficus* sp., and *Macaranga* sp. (Akoi, 2004; Husar, 1978; Keith‐Diagne, 2014; Powell, 1996; Reeves et al., 1988). In this study, we recorded 15 new consumed plant groups that were not previously reported in the literature. The newly reported plants include *Cynodon* sp., *Hypperhenia* sp., *Pycreus lanceolata*, *Remirea maritima*, *Eremospatha macrocarpa*, *Centrosema pubescens*, *Milletia mannii*, *Albizia* sp., *Platostoma* sp., *Acanthospermum* sp., *Calophyllum inophyllum*, *Canthium ciliatum*, *Ficus copreifolia*, *Ficus rubiginosa*, *and Ascolepis* sp.

#### 3.2.1 Manatee diet by location

The analysis of food plant composition from manatee feces showed that there was a moderate but significant difference between location (*R* = 0.18, *p *< .001). *A posteriori* pairwise comparison showed that only the difference in diet composition between Lake Ossa and Sanaga Estuary was significant (*p *< .001), with *E. pyramidalis*, *Cyperus* sp., and the Unidentified 13 morphospecies showing the greatest contribution to the dissimilarity between the two locations (41.5%, 27.2%, and 10.8%, respectively). The overall average dissimilarity between locations was 66.9%.

The Kruskal–Wallis analysis showed significant differences in the abundance of the five dominant plant species taken separately across all locations (Table S2). *Echinochloa pyramidalis* was the dominant species in all locations but was more abundant in feces from Lake Ossa (mean = 63.2%, *SD* = 33.34) and Upper Sanaga (mean = 59.7%, *SD* = 20.0); while *E. macrocarpa* was abundant only in Lake Tissongo (mean = 29.7%, *SD* = 35.3); *Cyperus* sp. was very abundant in the Sanaga Estuary (mean = 29.9%, *SD* = 44.12). Unidentified 13, a mostly filamentous plant, was only present and abundant in the Sanaga Estuary (mean = 12.1%, *SD* = 27.9), and *Rynchospora corymbosa* was most abundant in Lake Ossa (mean = 8.3%, *SD* = 12.9).

#### 3.2.2 Manatee diet by season in Lake Ossa

A total of 60 fecal samples collected in Lake Ossa were analyzed, including 29 collected during the high‐water season and 31 during the low‐water season. The analysis of diet composition in manatee feces indicated that there was a moderate but significant difference between seasons in Lake Ossa (*R* = 0.18, *p* = .00001). A total of 22 and 18 different plants were recorded in the fecal samples collected in Lake Ossa during the high‐ and low‐water seasons, respectively. The major contributors to the dissimilarity between seasons in Lake Ossa were *E. pyramidalis* (47.9%), *Cyperus* sp. (15.8%), and *R*. *corymbosa* (12.6%). The average dissimilarity between seasons was 53.2%.

The comparison of the abundance of individual dominant plants by season shows that only *E. pyramidalis* (*p* = .0001) and *Cyperus* sp. (*p* = .007) were significantly different across seasons (Table S3). *Echinochloa pyramidalis* fragments were two times more abundant in manatee feces during the low‐water season (mean = 81.1%, *SD* = 21.6) than the in high‐water season (mean = 44.2%, *SD* = 33.1). *Cyperus* sp. was 40 times more abundant in manatee feces during the high‐water season (mean = 15.3%, *SD* = 24.8) than during the low‐water season (mean = 0.4%, *SD* = 1.48; Figure 2c). Figure S4 gives a clear visualization of the manatee diet composition profile between the low‐ and high‐water seasons.

## 4 DISCUSSION

The high plant diversity along the shorelines (*H* = 3.52, 160 morphospecies) of the Sanaga River Watershed is characteristic of tropical areas of southern Cameroon that are considered to have the richest flora in continental tropical Africa (Myers, 1988). This high diversity of aquatic plant species likely provides a broad array of food options for the African manatee. Yet, plant species composition was highly variable among the four study locations, reflecting the difference in habitat type, water quality and salinity among those locations.

Lake Ossa and the Sanaga Estuary were the most dissimilar in plant composition, which is unsurprising because the distance between the two is the greatest (40 km) among locations. Also, Lake Ossa is purely freshwater while the Sanaga Estuary is brackish water that is under the influence of the tides. The manatee diet composition in these two locations was the most dissimilar. This suggests that manatees are opportunistically feeding on the most available plants in each location. Similar observations on the influence of the local vegetation on manatee diet have been reported among African manatee populations in the Ivory Coast (Akoi, 2004), Amazonian manatees (Guterres‐Pazin et al., 2014), Antillean manatee in Belize (Allen et al., 2018), and the Florida manatee (Alves‐Stanley et al., 2010).

Each manatee fecal sample contained between one and nine unique plant species. Similar results have been observed in Antillean manatees (Allen et al., 2018) and Amazonian manatees (Colares & Colares, 2002; Guterres‐Pazin et al., 2014). This further suggests that the African manatee is likely opportunistic in their diet, similar to the Amazonian and the West Indian manatee (Hartman, 1979; Marsh et al., 2011). This also might reflect the relatively high plant diversity of the area, as manatees often visit multiple sites in a single feeding bout (Akoi, 2004). We found that the diet proportions often closely reflected the plant composition of the area, which further suggests that the manatees are largely eating what is available. For example, *Echinochloa pyramidalis* appeared to be the most consumed plant species in this study, which contributed to 54% of the species diet in the study area, while making up 47% of the shoreline plants.

However, this does not appear to hold in some cases. In the Sanaga Estuary, *E. pyramidalis* represented only 3.5% of the shoreline vegetation, while it was the most observed plant in the feces (37%). It is possible that feces collected in the Sanaga Estuary were transported from the Upper Sanaga River by the downstream water flow, or that manatees themselves fed in the Upper Sanaga River where *E. pyramidalis* is more abundant before moving down to the estuary where they have deeper water to rest. It is also possible that manatees in the Sanaga River Watershed have some preference for *E. pyramidalis*, which was the most represented food in the diet at both the Sanaga Estuary and Lake Tissongo, despite the fact that it is not the dominant shoreline plant species. Thus, perhaps the African manatee prefers some plant species over others, but is opportunistic when food choices are limited.

Despite the abundance of fish and mollusk species in the Sanaga River Watershed, no fragment of fish or mollusk was present in any fecal sample examined in this study. This result does not corroborate local fishermen's reports of manatees eating fish and mollusks (Takoukam Kamla, 2012). It also fails to corroborate previous findings, indicating that 6–7% of the diet of African manatee in Gabon and 19–24% in Senegal was made up of hermit crabs (Keith‐Diagne, 2014). The diet reported here is likely biased in favor of the highly fibrous plants, which can easily survive passage through the digestive tract (Hurst & Beck, 1988). Differential digestion also could render some plant species unidentifiable. More comprehensive stable isotope studies from the Sanaga River will be crucial to our understanding of the level of omnivory in the diet of African manatees in this location.

Despite the abundance of mangrove in the Sanaga Estuary, no *Rhizophora* sp. fragment was recorded in the feces, whereas the plant has been reported as an African manatee diet item in several countries including Cameroon (Husar, 1978; Keith‐Diagne, 2014; Powell, 1996). The absence of *Rhizophora* sp. in the diet of manatee in this study could be due to the abundance of grasses in the mangrove area which are more available and perhaps more nutrient‐rich for the manatees than the mangrove leaves hanging over the estuary edges. Manatees are, in general, grazers rather than browsers (Marsh et al., 2011). It is also possible that our sampling period and effort were not representative enough to capture seasonal manatee feeding on mangrove.

Manatee diets here consisted of 96% emergent grasses, which occurred in all fecal samples analyzed—consistent with previous work in the Ivory Coast (Akoi, 2004). Floating plant species represented an insignificant fraction of our fecal samples, and no submerged plant fragments were detected. The absence of submerged aquatic plants in the entire study area could be associated with the high turbidity and low transparency of water, preventing light from reaching the bottom to sustain plant growth. This may also be due to recent increases in nutrient enrichment of the lake that might have been caused by the construction of the largest reservoir dam of the country (Lom‐Pangar), upstream on the Sanaga River.

The seasonal effect on the manatee diet was explored only in Lake Ossa because of the low fecal sample size for the low‐water season in the other locations, further highlighting that Lake Ossa is a refuge for manatees during the low‐water season (Takoukam Kamla, 2019). There was a significant difference in manatee diet composition between the low‐ and high‐water seasons in Lake Ossa. This result is unsurprising as the shoreline plant survey showed that the number of species available during the high season was nearly threefold greater than that of the low‐water season. Indeed, manatee diets reflected this difference as more plant species were observed in feces during the high‐water season.

The abundance of food plants during the high‐water season may be partially responsible for the seasonal manatee breeding activity. African manatee mating herd behavior primarily starts during the beginning of the rainy season in June/July (Cadenat, 1957; Dodman et al., 2008; Powell, 1996). The African manatee may have adapted to synchronize their breeding period with the seasonal variation in the availability of food so that calves are born during rising water when females have access to a greater amount and better quality of forage necessary to increase fat reserves (or energy reserves) to support gestation and lactation.

As water levels rise and the grass plains of the lake are flooded, manatees gain access to more diverse and abundant plant species. When the water level drops during the dry season, manatee food availability is reduced and limited to floating or semi‐floating species. Floating plant species were scarce in Lake Ossa, and *E. pyramidalis* was the major emergent species. Therefore, it is not surprising that the manatee diet in Lake Ossa was highly dominated by *E. pyramidalis* (81%) during the low‐water season whereas it only represented 44% of the diet during the high‐water season.

Due to this lower food availability, relative to the high‐water season, the manatee population in Lake Ossa may leave the lake and move to the Sanaga Estuary where they can take advantage of the high tide for access to shoreline plants. Indeed, interview surveys among 144 fishermen in the Sanaga River Watershed indicated that manatees are more frequently seen in the Sanaga River estuary during the low‐water (dry) season than during the high‐water (rainy) season (Takoukam Kamla, 2012).

## 5 CONCLUSIONS

This work documents the current, but changing, state of plant availability in the Sanaga River Watershed and the African manatee diet in Cameroon for the first time. This information fills an existing knowledge gap and can play a critical role in successfully informing management of the species and these protected areas. For successful long‐term protection of the African manatee and the aquatic ecosystems within the Sanaga River watershed, it is critical to limit nutrient enrichment, and subsequent eutrophication, and to stop the spread of invasive plants species.

## CONFLICT OF INTEREST

The authors declare no conflict of interest.

## AUTHOR CONTRIBUTIONS

**Aristide Takoukam Kamla:** Conceptualization (lead); data curation (lead); formal analysis (lead); funding acquisition (lead); investigation (lead); methodology (lead); resources (lead); visualization (lead); writing‐original draft (lead); writing‐review & editing (supporting). **Dylan G. E. Gomes:** Data curation (supporting); visualization (supporting); writing‐original draft (supporting); writing‐review & editing (lead). **Cathy A. Beck:** Conceptualization (supporting); funding acquisition (supporting); methodology (lead); resources (supporting); supervision (lead); validation (lead); writing‐review & editing (supporting). **Lucy W. Keith‐Diagne:** Conceptualization (supporting); funding acquisition (supporting); resources (supporting); supervision (supporting); writing‐review & editing (supporting). **Margaret E. Hunter:** Conceptualization (supporting); funding acquisition (supporting); resources (supporting); supervision (supporting); writing‐review & editing (supporting). **Ruth Francis‐Floyd:** Conceptualization (supporting); funding acquisition (supporting); resources (supporting); supervision (supporting); writing‐review & editing (supporting). **Robert K. Bonde:** Conceptualization (supporting); funding acquisition (supporting); methodology (lead); resources (supporting); supervision (lead); validation (lead); writing‐review & editing (supporting).

### OPEN RESEARCH BADGES

This article has earned an Open Data, for making publicly available the digitally‐shareable data necessary to reproduce the reported results. The data is available at https://doi.org/10.5281/zenodo.4560427.

## Supporting information

Appendix S1

## Data Availability

All data are available on Zenodo (https://doi.org/10.5281/zenodo.4560427).
