# Supplementary material for: Diet composition of the African manatee: Spatial and temporal variation within the Sanaga River Watershed, Cameroon
Source: Ecol Evol. 2021 Nov 2;11(22):15833–45. doi: 10.1002/ece3.8254 (PMC8601922; doi:10.1002/ece3.8254)
Supplement: Supplementary file 1 — Appendix S1 [file ECE3-11-15833-s001.docx]

Supplementary Appendix:

Diet composition of the African manatee: spatial and temporal variation

within the downstream Sanaga River watershed, Cameroon

Table S1. List of plant group, family and species by location, number of plots surveyed (in the brackets), and their relative abundance in percentage.

| Plant type | Sanaga Estuary (n=80) | Upper Sanaga (n=146) | Lake Tissongo (n=64) | Lake Ossa (n=668) | All sites (n=958) |
| --- | --- | --- | --- | --- | --- |
| **Emergent macrophytes** | **37.44** | **69.53** | **90.63** | **85.49** | **70.77** |
| **Amaranthaceae** | **0.00** | **0.17** | **0.00** | **0.01** | **0.05** |
| *Amaranthus esculentus* | 0.00 | 0.07 | 0.00 | 0.00 | 0.02 |
| *Cyathula prostrata* | 0.00 | 0.10 | 0.00 | 0.01 | 0.03 |
| **Amaryllidaceae** | **0.00** | **0.00** | **0.00** | **0.01** | **0.00** |
| *Allium sp.* | 0.00 | 0.00 | 0.00 | 0.01 | 0.00 |
| **Araceae** | **1.38** | **0.55** | **0.78** | **0.16** | **0.72** |
| *Colocassia esculentus* | 0.00 | 0.55 | 0.00 | 0.00 | 0.14 |
| *Cyrtosperma merkusii* | 0.00 | 0.00 | 0.00 | 0.03 | 0.01 |
| *Cyrtosperma senegalense* | 1.38 | 0.00 | 0.78 | 0.13 | 0.57 |
| **Arecaceae** | **3.38** | **0.00** | **37.97** | **6.77** | **12.03** |
| *Calamus acidus* | 0.00 | 0.00 | 0.00 | 0.81 | 0.20 |
| *Eremospatha macrocarpa* | 0.63 | 0.00 | 32.34 | 2.07 | 8.76 |
| *Laccosperma robustum* | 0.00 | 0.00 | 0.00 | 2.00 | 0.50 |
| *Laccosperma secundiflorum* | 0.00 | 0.00 | 5.63 | 1.89 | 1.88 |
| *Nypa fruticans* | 2.50 | 0.00 | 0.00 | 0.00 | 0.63 |
| *Raphia sp.* | 0.25 | 0.00 | 0.00 | 0.00 | 0.06 |
| **Asteraceae** | **0.38** | **3.18** | **0.00** | **0.37** | **0.98** |
| *Ageratum conyzoides* | 0.00 | 1.37 | 0.00 | 0.21 | 0.39 |
| *Chromolaena odorata* | 0.00 | 0.05 | 0.00 | 0.00 | 0.01 |
| *Eclipta prostrata* | 0.13 | 0.89 | 0.00 | 0.12 | 0.28 |
| *Melanthera scandens* | 0.25 | 0.21 | 0.00 | 0.03 | 0.12 |
| *Mikania micrantha* | 0.00 | 0.66 | 0.00 | 0.00 | 0.17 |
| Unknown Asteraceae | 0.00 | 0.00 | 0.00 | 0.01 | 0.00 |
| **Athyriaceae** | **0.00** | **0.74** | **0.00** | **0.76** | **0.37** |
| *Diplazium sammatii* | 0.00 | 0.74 | 0.00 | 0.76 | 0.37 |
| **Boraginaceae** | **0.00** | **1.03** | **0.00** | **0.06** | **0.27** |
| *Heliotropium indicum* | 0.00 | 1.03 | 0.00 | 0.06 | 0.27 |
| **Calophyllaceae** | **1.23** | **0.00** | **0.00** | **0.00** | **0.31** |
| *Calophyllum inophyllum* | 1.23 | 0.00 | 0.00 | 0.00 | 0.31 |
| **Cleomaceae** | **0.00** | **1.14** | **0.00** | **0.13** | **0.32** |
| *Cleome ciliata* | 0.00 | 0.03 | 0.00 | 0.00 | 0.01 |
| *Cleome rudidosperma* | 0.00 | 0.14 | 0.00 | 0.03 | 0.04 |
| *Cleome spinosa* | 0.00 | 0.97 | 0.00 | 0.10 | 0.27 |
| **Commelinaceae** | **1.25** | **2.14** | **1.88** | **0.47** | **1.43** |

Table S1. Continued

| Plant type | Sanaga Estuary (n=80) | Upper Sanaga (n=146) | Lake Tissongo (n=64) | Lake Ossa (n=668) | All sites (n=958) |
| --- | --- | --- | --- | --- | --- |
| *Commelina benghalensis* | 1.25 | 2.14 | 1.88 | 0.47 | 1.43 |
| **Convolvulaceae** | **1.30** | **14.78** | **0.00** | **1.80** | **4.47** |
| *Hewittia sublobata* | 0.00 | 0.12 | 0.00 | 0.00 | 0.03 |
| *Ipomoea alba* | 0.00 | 9.35 | 0.00 | 1.59 | 2.73 |
| *Ipomoea batatas* | 0.00 | 0.14 | 0.00 | 0.00 | 0.03 |
| *Ipomoea involucrata* | 0.69 | 2.47 | 0.00 | 0.01 | 0.79 |
| *Ipomoea mauritiana* | 0.13 | 0.07 | 0.00 | 0.00 | 0.05 |
| *Ipomoea preussii* | 0.00 | 0.14 | 0.00 | 0.00 | 0.03 |
| *Ipomoea quamoclit* | 0.00 | 0.07 | 0.00 | 0.01 | 0.02 |
| *Ipomoea sp.* | 0.25 | 1.82 | 0.00 | 0.15 | 0.55 |
| *Ipomoea triloba* | 0.24 | 0.62 | 0.00 | 0.03 | 0.22 |
| **Costaceae** | **0.00** | **0.23** | **0.00** | **0.01** | **0.06** |
| *Costus afer* | 0.00 | 0.23 | 0.00 | 0.01 | 0.06 |
| **Cucurbitaceae** | **0.00** | **1.21** | **0.00** | **0.00** | **0.30** |
| *Cucumis melo* | 0.00 | 0.55 | 0.00 | 0.00 | 0.14 |
| *Cucumis moschata* | 0.00 | 0.17 | 0.00 | 0.00 | 0.04 |
| *Luffa aegyptiaca* | 0.00 | 0.34 | 0.00 | 0.00 | 0.09 |
| *Momordica charantia* | 0.00 | 0.14 | 0.00 | 0.00 | 0.04 |
| **Cyperaceae** | **1.64** | **0.00** | **2.34** | **5.04** | **2.26** |
| *Cyperus haspan* | 0.50 | 0.00 | 0.78 | 1.29 | 0.64 |
| *Cyperus papyrus* | 1.14 | 0.00 | 0.00 | 0.00 | 0.28 |
| *Fuirena umbellata* | 0.00 | 0.00 | 1.56 | 1.89 | 0.86 |
| *Pycreus lanceolatus* | 0.00 | 0.00 | 0.00 | 1.86 | 0.47 |
| *Rynchospora corymbosa** | na | na | na | na | na |
| **Dioscoreaceae** | **0.00** | **0.10** | **0.00** | **0.00** | **0.03** |
| *Dioscorea cayenensis* | 0.00 | 0.10 | 0.00 | 0.00 | 0.03 |
| **Fabaceae** | **1.38** | **3.33** | **0.16** | **0.59** | **1.36** |
| *Aechynomene sensitiva* | 0.00 | 0.00 | 0.00 | 0.03 | 0.01 |
| *Aeschynomene indica* | 0.63 | 0.00 | 0.00 | 0.09 | 0.18 |
| *Aeschynomene sensitiva* | 0.00 | 0.00 | 0.00 | 0.07 | 0.02 |
| *Calopogonium mucunoides* | 0.00 | 0.03 | 0.16 | 0.03 | 0.06 |
| *Canavalia rosea* | 0.06 | 0.00 | 0.00 | 0.00 | 0.02 |
| *Centrosema pubescens* | 0.00 | 0.24 | 0.00 | 0.01 | 0.06 |
| *Pueraria phaseoloides* | 0.38 | 2.64 | 0.00 | 0.04 | 0.76 |
| *Vigna lutea* | 0.00 | 0.00 | 0.00 | 0.10 | 0.03 |
| *Vigna radiata* | 0.31 | 0.41 | 0.00 | 0.22 | 0.24 |
| **Hydroleaceae** | **0.00** | **0.00** | **0.00** | **0.03** | **0.01** |
| *Hydrolea sp.* | 0.00 | 0.00 | 0.00 | 0.03 | 0.01 |
| **Lamiaceae** | **0.00** | **1.64** | **0.00** | **0.37** | **0.50** |
| *Hyptis lanceolata* | 0.00 | 1.47 | 0.00 | 0.31 | 0.45 |

Table S1. Continued

| Plant type | Sanaga Estuary (n=80) | Upper Sanaga (n=146) | Lake Tissongo (n=64) | Lake Ossa (n=668) | All sites (n=958) |
| --- | --- | --- | --- | --- | --- |
| *Leonotis sp.* | 0.00 | 0.07 | 0.00 | 0.04 | 0.03 |
| *Solenostemon monostachyus* | 0.00 | 0.10 | 0.00 | 0.01 | 0.03 |
| **Liliaceae** | **0.00** | **0.00** | **1.56** | **0.00** | **0.39** |
| Unknown Liliaceae | 0.00 | 0.00 | 1.56 | 0.00 | 0.39 |
| **Malvaceae** | **1.50** | **1.34** | **0.00** | **0.06** | **0.72** |
| *Abutilon sp.* | 0.25 | 0.68 | 0.00 | 0.00 | 0.23 |
| *Clappertonia sp.* | 0.00 | 0.00 | 0.00 | 0.03 | 0.01 |
| *Corchorus olitorius* | 0.00 | 0.07 | 0.00 | 0.00 | 0.02 |
| *Melochia corchorifolia* | 0.00 | 0.27 | 0.00 | 0.01 | 0.07 |
| *Triumfetta cordifolia* | 0.00 | 0.17 | 0.00 | 0.00 | 0.04 |
| Unknown Malvaceae | 1.25 | 0.00 | 0.00 | 0.00 | 0.31 |
| *Urena lobata* | 0.00 | 0.14 | 0.00 | 0.01 | 0.04 |
| **Marantaceae** | **1.19** | **0.48** | **0.00** | **0.01** | **0.42** |
| *Haumania danckelmaniana* | 0.00 | 0.00 | 0.00 | 0.01 | 0.00 |
| *Marantochloa sp.* | 1.19 | 0.48 | 0.00 | 0.00 | 0.42 |
| **Melastomataceae** | **2.44** | **2.05** | **27.11** | **10.72** | **10.58** |
| *Dissotis erecta* | 2.44 | 1.91 | 27.11 | 5.13 | 9.15 |
| *Dissotis falcipila* | 0.00 | 0.14 | 0.00 | 5.57 | 1.43 |
| *Dissotis rontondifolia* | 0.00 | 0.00 | 0.00 | 0.01 | 0.00 |
| **Nephrolepidaceae** | **0.00** | **0.21** | **0.00** | **0.00** | **0.05** |
| *Nephrolepis biserrata* | 0.00 | 0.21 | 0.00 | 0.00 | 0.05 |
| **Onagraceae** | **0.13** | **0.63** | **0.00** | **4.44** | **1.30** |
| *Ludwigia decurrens* | 0.13 | 0.17 | 0.00 | 0.04 | 0.08 |
| *Ludwigia hyssopifolia* | 0.00 | 0.46 | 0.00 | 1.17 | 0.41 |
| *Ludwigia Stolonifera* | 0.00 | 0.00 | 0.00 | 3.23 | 0.81 |
| **Orchidaceae** | **0.00** | **0.07** | **0.00** | **0.01** | **0.02** |
| *Ansellia africana* | 0.00 | 0.07 | 0.00 | 0.01 | 0.02 |
| **Oxalidaceae** | **0.00** | **0.00** | **0.00** | **0.04** | **0.01** |
| *Oxalis barrelieri* | 0.00 | 0.00 | 0.00 | 0.04 | 0.01 |
| **Pandanaceae** | **6.25** | **0.00** | **0.00** | **0.00** | **1.56** |
| *Pandanus candelabrum* | 6.25 | 0.00 | 0.00 | 0.00 | 1.56 |
| **Passifloraceae** | **0.00** | **0.31** | **0.00** | **0.00** | **0.08** |
| *Passiflora foetida* | 0.00 | 0.31 | 0.00 | 0.00 | 0.08 |
| **Poaceae** | **10.56** | **30.18** | **18.67** | **50.46** | **27.47** |
| *Acroceras zizanioides* | 1.25 | 1.71 | 0.00 | 1.12 | 1.02 |
| *Bambousa vulgaris* | 0.00 | 0.03 | 0.00 | 0.01 | 0.01 |
| *Cynodon dactylon* | 0.00 | 0.07 | 0.00 | 0.10 | 0.04 |
| *Echinochloa pyramidalis* | 3.25 | 15.88 | 16.80 | 46.95 | 20.72 |
| *Leersia hexandra* | 0.25 | 0.27 | 0.00 | 0.45 | 0.24 |
| *Melinis sp.* | 0.00 | 0.00 | 1.88 | 0.18 | 0.51 |

Table S1. Continued

| Plant type | Sanaga Estuary (n=80) | Upper Sanaga (n=146) | Lake Tissongo (n=64) | Lake Ossa (n=668) | All sites (n=958) |
| --- | --- | --- | --- | --- | --- |
| *Panicum maximum* | 0.00 | 0.89 | 0.00 | 0.04 | 0.23 |
| *Paspalum conjugatum* | 0.00 | 0.14 | 0.00 | 0.00 | 0.03 |
| *Paspalum dilatatum* | 0.00 | 0.00 | 0.00 | 0.11 | 0.03 |
| *Paspalum polystachyum* | 0.00 | 0.96 | 0.00 | 0.61 | 0.39 |
| *Paspalum purpureum* | 0.00 | 1.71 | 0.00 | 0.37 | 0.52 |
| *Paspalum sp.* | 0.00 | 0.10 | 0.00 | 0.04 | 0.04 |
| *Pennisetum purpureum* | 5.81 | 6.61 | 0.00 | 0.32 | 3.19 |
| *Sacciolepsis africana* | 0.00 | 0.00 | 0.00 | 0.07 | 0.02 |
| *Setaria barbata* | 0.00 | 0.32 | 0.00 | 0.01 | 0.08 |
| *Sorghum arundinaceum* | 0.00 | 1.47 | 0.00 | 0.06 | 0.38 |
| **Polygonaceae** | **0.75** | **3.13** | **0.00** | **2.11** | **1.50** |
| *Polygonum lanceolata* | 0.00 | 0.00 | 0.00 | 0.61 | 0.15 |
| *Polygonum lanigerum* | 0.38 | 2.33 | 0.00 | 0.90 | 0.90 |
| *polygonum salicifolium* | 0.38 | 0.80 | 0.00 | 0.61 | 0.45 |
| *Polypodiidae* | 0.00 | 0.00 | 0.00 | 0.12 | 0.03 |
| *Leptosporangiate fern* | 0.00 | 0.00 | 0.00 | 0.12 | 0.03 |
| **Proteaceae** | **0.00** | **0.01** | **0.00** | **0.00** | **0.00** |
| *Leucospermum sp.* | 0.00 | 0.01 | 0.00 | 0.00 | 0.00 |
| **Pteridaceae** | **2.50** | **0.00** | **0.00** | **0.00** | **0.63** |
| *Acrostichum aureum* | 2.50 | 0.00 | 0.00 | 0.00 | 0.63 |
| **Rubiaceae** | **0.06** | **0.22** | **0.16** | **0.90** | **0.34** |
| *Galium sp* | 0.00 | 0.00 | 0.00 | 0.01 | 0.00 |
| *Oldenlandia corymbosa* | 0.00 | 0.00 | 0.16 | 0.06 | 0.05 |
| *Oldenlandia diffusa* | 0.00 | 0.02 | 0.00 | 0.83 | 0.21 |
| *Pentodon pentandrus* | 0.00 | 0.20 | 0.00 | 0.00 | 0.05 |
| *Stipularia africana* | 0.06 | 0.00 | 0.00 | 0.00 | 0.02 |
| **Solanaceae** | **0.00** | **0.10** | **0.00** | **0.00** | **0.03** |
| *Physalis angulata* | 0.00 | 0.07 | 0.00 | 0.00 | 0.02 |
| *Solanum nigrum* | 0.00 | 0.03 | 0.00 | 0.00 | 0.01 |
| **Verbenaceae** | **0.00** | **0.34** | **0.00** | **0.01** | **0.09** |
| *Stachytarpheta jamaicensis* | 0.00 | 0.34 | 0.00 | 0.01 | 0.09 |
| **Vitaceae** | **0.15** | **0.23** | **0.00** | **0.01** | **0.10** |
| *Cayratia ibuensis* | 0.16 | 0.23 | 0.00 | 0.01 | 0.09 |
| **Free-floating macrophytes** | **8.31** | **4.21** | **0.00** | **4.58** | **4.28** |
| **Araceae** | **0.00** | **0.21** | **0.00** | **0.15** | **0.09** |
| *Pistia stratoides* | 0.00 | 0.21 | 0.00 | 0.15 | 0.09 |
| **Azollaceae** | **0.00** | **0.00** | **0.00** | **0.36** | **0.09** |
| *Azolla africana* | 0.00 | 0.00 | 0.00 | 0.36 | 0.09 |
| **Lemnaceae** | **0.00** | **0.21** | **0.00** | **0.18** | **0.10** |
| *Lemna minor* | 0.00 | 0.21 | 0.00 | 0.18 | 0.10 |

Table S1. Continued

| Plant type | Sanaga Estuary (n=80) | Upper Sanaga (n=146) | Lake Tissongo (n=64) | Lake Ossa (n=668) | All sites (n=958) |
| --- | --- | --- | --- | --- | --- |
| **Lentibulariaceae** | **0.00** | **0.00** | **0.00** | **0.70** | **0.17** |
| *Utricularia vulgaris* | 0.00 | 0.00 | 0.00 | 0.70 | 0.17 |
| **Nymphaeaceae** | **0.00** | **0.00** | **0.00** | **1.04** | **0.26** |
| *Nymphaea lotus* | 0.00 | 0.00 | 0.00 | 1.04 | 0.26 |
| **Pontederiaceae** | **8.31** | **3.80** | **0.00** | **0.00** | **3.03** |
| *Eichornia crassipes* | 8.31 | 3.80 | 0.00 | 0.00 | 3.03 |
| **Salviniaceae** | **0.00** | **0.00** | **0.00** | **2.15** | **0.54** |
| *Salvinia molesta* | 0.00 | 0.00 | 0.00 | 2.15 | 0.54 |
| **Shrubs** | **22.56** | **11.47** | **8.28** | **5.38** | **11.92** |
| **Apocynaceae** | **0.00** | **0.00** | **0.00** | **0.27** | **0.07** |
| *Allamanda cathartica* | 0.00 | 0.00 | 0.00 | 0.01 | 0.00 |
| *Landolphia senegalensis* | 0.00 | 0.00 | 0.00 | 0.25 | 0.06 |
| **Arecaceae** | **0.00** | **0.00** | **0.00** | **0.01** | **0.00** |
| *Elaeis guineensis* | 0.00 | 0.00 | 0.00 | 0.01 | 0.00 |
| **Dilleniaceae** | **0.00** | **0.00** | **0.00** | **0.07** | **0.02** |
| *Tetracera macrophylla* | 0.00 | 0.00 | 0.00 | 0.07 | 0.02 |
| **Euphorbiaceae** | **11.50** | **6.44** | **6.72** | **0.53** | **6.30** |
| *Alchornea cordifolia* | 10.56 | 4.32 | 6.72 | 0.53 | 5.53 |
| *Macaranga sp.* | 0.94 | 0.00 | 0.00 | 0.00 | 0.23 |
| *Mallotus oppositifolius* | 0.00 | 0.58 | 0.00 | 0.00 | 0.15 |
| *Manihot esculenta* | 0.00 | 1.54 | 0.00 | 0.00 | 0.39 |
| **Fabaceae** | **0.00** | **1.61** | **0.00** | **0.10** | **0.43** |
| *Millettia macrophylla* | 0.00 | 0.96 | 0.00 | 0.10 | 0.27 |
| *Millettia sanagana* | 0.00 | 0.65 | 0.00 | 0.00 | 0.16 |
| **Malvaceae** | **0.50** | **0.82** | **0.00** | **0.16** | **0.37** |
| *Glyphaea brevis* | 0.50 | 0.82 | 0.00 | 0.16 | 0.37 |
| **Phyllanthaceae** | **5.13** | **0.48** | **1.56** | **0.34** | **1.88** |
| *Phyllanthus amarus* | 0.00 | 0.38 | 0.00 | 0.06 | 0.11 |
| *Phyllanthus reticulatus* | 0.00 | 0.03 | 0.00 | 0.08 | 0.03 |
| *Uapaca guineensis* | 5.00 | 0.07 | 1.56 | 0.16 | 1.70 |
| *Uapaca mole* | 0.13 | 0.00 | 0.00 | 0.03 | 0.04 |
| **Rubiaceae** | **1.25** | **0.68** | **0.00** | **3.85** | **1.45** |
| *Canthium angustifolium* | 1.25 | 0.00 | 0.00 | 0.01 | 0.31 |
| *Canthium ciliatum* | 0.00 | 0.68 | 0.00 | 3.82 | 1.13 |
| *Nauclea pobeguinii* | 0.00 | 0.00 | 0.00 | 0.03 | 0.01 |
| **Sapindaceae** | **4.19** | **1.44** | **0.00** | **0.03** | **1.41** |
| *Allophylus africanus* | 0.31 | 0.00 | 0.00 | 0.00 | 0.08 |
| *Allophylus bullatus* | 0.00 | 0.14 | 0.00 | 0.00 | 0.03 |
| *Paullinia pinnata* | 3.88 | 1.30 | 0.00 | 0.03 | 1.30 |
| **Trees** | **31.69** | **14.78** | **1.09** | **4.55** | **13.03** |
| **Annonaceae** | **1.19** | **0.00** | **0.00** | **0.58** | **0.44** |

Table S1. Continued

| Plant type | Sanaga Estuary (n=80) | Upper Sanaga (n=146) | Lake Tissongo (n=64) | Lake Ossa (n=668) | All sites (n=958) |
| --- | --- | --- | --- | --- | --- |
| *Cleistopholis patens* | 1.19 | 0.00 | 0.00 | 0.00 | 0.30 |
| *Xylopia sp.* | 0.00 | 0.00 | 0.00 | 0.55 | 0.14 |
| *Xylopia sp.2* | 0.00 | 0.00 | 0.00 | 0.03 | 0.01 |
| **Clusiaceae** | **0.00** | **0.00** | **0.00** | **0.09** | **0.02** |
| *Symphonia globulifera* | 0.00 | 0.00 | 0.00 | 0.09 | 0.02 |
| **Combretaceae** | **0.94** | **0.48** | **0.00** | **0.00** | **0.35** |
| *Combretum zenkeri* | 0.31 | 0.48 | 0.00 | 0.00 | 0.20 |
| *Terminalia catappa* | 0.63 | 0.00 | 0.00 | 0.00 | 0.16 |
| **Fabaceae** | **0.00** | **0.79** | **0.47** | **1.32** | **0.64** |
| *Acacia sp.* | 0.00 | 0.79 | 0.00 | 0.15 | 0.23 |
| *Dalbergia sp.* | 0.00 | 0.00 | 0.00 | 1.17 | 0.29 |
| *Guibourtia demeusei* | 0.00 | 0.00 | 0.47 | 0.00 | 0.12 |
| **Gentianaceae** | **1.25** | **0.00** | **0.63** | **0.01** | **0.47** |
| *Anthocleista djalonensis* | 0.00 | 0.00 | 0.63 | 0.01 | 0.16 |
| *Anthocleista liebrechtsiana* | 1.25 | 0.00 | 0.00 | 0.00 | 0.31 |
| **Meliaceae** | **2.88** | **0.68** | **0.00** | **0.15** | **0.93** |
| *Trichilia emetica* | 2.88 | 0.68 | 0.00 | 0.15 | 0.93 |
| **Moraceae** | **3.25** | **10.88** | **0.00** | **1.55** | **3.92** |
| *Ficus benghalensis* | 0.00 | 0.00 | 0.00 | 0.37 | 0.09 |
| *Ficus capreifolia* | 0.00 | 5.21 | 0.00 | 1.03 | 1.56 |
| *Ficus mucuso* | 0.00 | 0.07 | 0.00 | 0.01 | 0.02 |
| *Ficus capreifolia* | 3.25 | 5.60 | 0.00 | 0.13 | 2.25 |
| **Rhizophoraceae** | **19.88** | **0.00** | **0.00** | **0.00** | **4.97** |
| *Rhizophora racemosa* | 19.88 | 0.00 | 0.00 | 0.00 | 4.97 |
| **Urticaceae** | **2.31** | **1.95** | **0.00** | **0.85** | **1.28** |
| *Cecropia obtusifolia* | 0.00 | 0.00 | 0.00 | 0.43 | 0.11 |
| *Myrianthus serratus* | 2.31 | 1.95 | 0.00 | 0.41 | 1.17 |

Table S2. Major identified diet plant species of African manatees surveyed by location of the downstream Sanaga River watershed. The stars represent the level of significance (determined using the Kruskal-Wallis test) in the frequency of each plant species between each pair-wise location. The second column indicates the locations for which significance was observed. Min = minimum, Max = maximum, SD = Standard deviation, Var = variance, SE = Standard error and CV = Coefficient of variation

| Location | Pair-wise significance | Mean | Min | Max | SD | Var | SE | CV |
| --- | --- | --- | --- | --- | --- | --- | --- | --- |
| **^a^Lake Ossa (n=60)** |  |  |  |  |  |  |  |  |
| *Echinochloa pyramidalis*** | c | 63.23 | 0 | 100.00 | 33.34 | 1111.65 | 4.34 | 0.53 |
| *Eremospatha macrocarpa**** | b,c,d | 3.40 | 0 | 28.00 | 6.37 | 40.53 | 0.83 | 1.87 |
| *Rynchospora corymbosa**** | b,c,d | 8.27 | 0 | 65.00 | 12.94 | 167.46 | 1.68 | 1.56 |
| *Cyperus* sp*.** | c | 7.61 | 0 | 74.00 | 18.80 | 353.28 | 2.45 | 2.47 |
| Unidentified 13*** | c | 0 | 0 | 0 | 0 | 0 | 0 |  |
| **^b^Lake Tissongo (n=11)** |  |  |  |  |  |  |  |  |
| *Echinochloa pyramidalis*** | - | 44.60 | 2.0 | 76.00 | 26.17 | 684.65 | 8.27 | 0.59 |
| *Eremospatha macrocarpa**** | a,c,d | 29.69 | 0 | 95.00 | 35.26 | 1243.10 | 11.15 | 1.19 |
| *Rynchospora corymbosa**** | a | 0.09 | 0 | 1.00 | 0.29 | 0.08 | 0.09 | 3.16 |
| *Cyperus* sp. | c | 0.55 | 0 | 6.00 | 1.72 | 2.98 | 0.55 | 3.16 |
| Unidentified 13*** | c | 0 | 0 | 0 | 0 | 0 | 0 |  |
| **^c^Estuary Sanaga (n=33)** |  |  |  |  |  |  |  |  |
| *Echinochloa pyramidalis*** | a | 37.14 | 0 | 100.00 | 40.06 | 1604.87 | 7.08 | 1.08 |
| *Eremospatha macrocarpa**** | a,b | 0.09 | 0 | 3.00 | 0.51 | 0.26 | 0.09 | 5.66 |
| *Rynchospora corymbosa**** | a | 2.58 | 0 | 33.00 | 6.49 | 42.06 | 1.15 | 2.52 |
| *Cyperus* sp.* | a,b | 29.94 | 0 | 100.00 | 44.12 | 1946.18 | 7.80 | 1.47 |
| Unidentified 13*** | a,b,d | 12.08 | 0 | 84.00 | 27.85 | 775.49 | 4.92 | 2.31 |
| **^d^Upper Sanaga (n=09)** |  |  |  |  |  |  |  |  |
| *Echinochloa pyramidalis*** | - | 59.67 | 30.20 | 88.60 | 20.02 | 400.61 | 7.08 | 0.34 |
| *Eremospatha macrocarpa**** | a,b | 0 | 0 | 0 | 0 | 0 | 0 |  |
| *Rynchospora corymbosa**** | a | 0 | 0 | 0 | 0 | 0 | 0 |  |
| *Cyperus* sp.* | - | 0.22 | 0 | 2.00 | 0.63 | 0.40 | 0.22 | 2.83 |
| Unidentified 13*** | c | 0 | 0 | 0.0 | 0.0 | 0.0 | 0.0 |  |

Table S3. Major identified diet plant species of African manatees surveyed by seasons in Lake Ossa. The stars represent the level of significance (determined using the Kruskal-Wallis test) in the frequency of each plant species between the low- and the high-water seasons. Min = minimum, Max = maximum, SD = Standard deviation, Var = variance, SE = Standard error and CV = Coefficient of variation.

| Season (water level) | Mean | Min | Max | SD | Var | SE | CV |
| --- | --- | --- | --- | --- | --- | --- | --- |
| **High water (n=29)** |  |  |  |  |  |  |  |
| *Echinochloa pyramidalis**** | 44.17 | 0 | 100.00 | 33.11 | 1096.02 | 6.26 | 0.75 |
| *Eremospatha macrocarpa* | 5.12 | 0 | 28.00 | 7.79 | 60.74 | 1.47 | 1.52 |
| *Leersia hexandra* | 6.97 | 0 | 71.00 | 15.38 | 236.57 | 2.91 | 2.21 |
| *Rynchospora corymbosa* | 10.01 | 0 | 65.00 | 14.58 | 212.66 | 2.76 | 1.46 |
| *Cyperus* sp.** | 15.34 | 0 | 74.00 | 24.75 | 612.73 | 4.68 | 1.61 |
| **Low water (n=31)** |  |  |  |  |  |  |  |
| *Echinochloa pyramidalis**** | 81.07 | 3 | 100.00 | 21.64 | 468.08 | 3.95 | 0.27 |
| *Eremospatha macrocarpa* | 1.79 | 0 | 16.00 | 4.03 | 16.27 | 0.74 | 2.25 |
| *Leersia hexandra* | 2.26 | 0 | 31.40 | 6.84 | 46.82 | 1.25 | 3.03 |
| *Rynchospora corymbosa* | 6.65 | 0 | 47.60 | 10.94 | 119.73 | 2.00 | 1.65 |
| *Cyperus* sp.** | 0.37 | 0 | 7.50 | 1.48 | 2.19 | 0.27 | 3.99 |


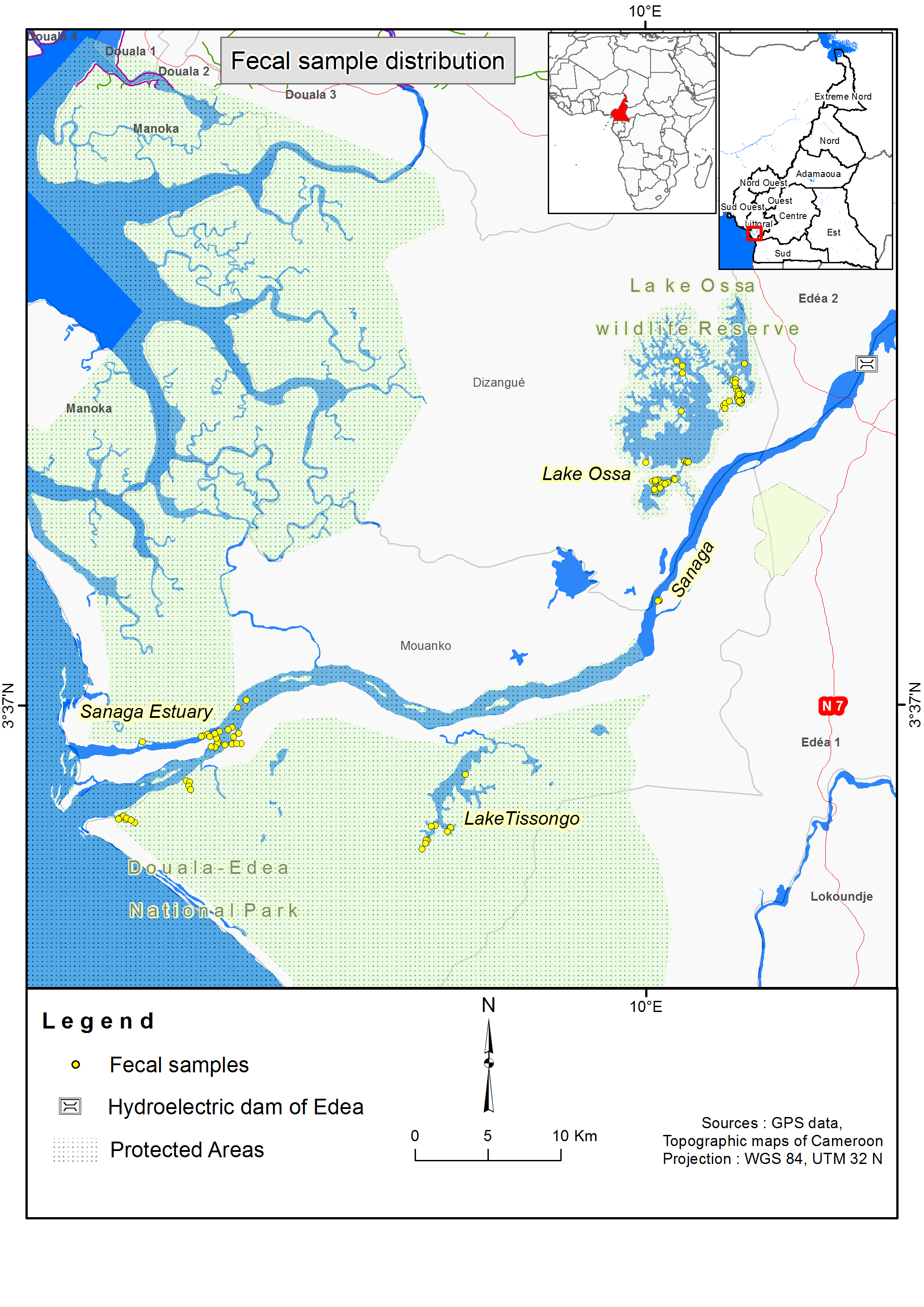


Figure S1. Map of the downstream Sanaga River watershed showing the spatial distribution of the 113 feces collected and used in this study. Collection sites are yellow circles.


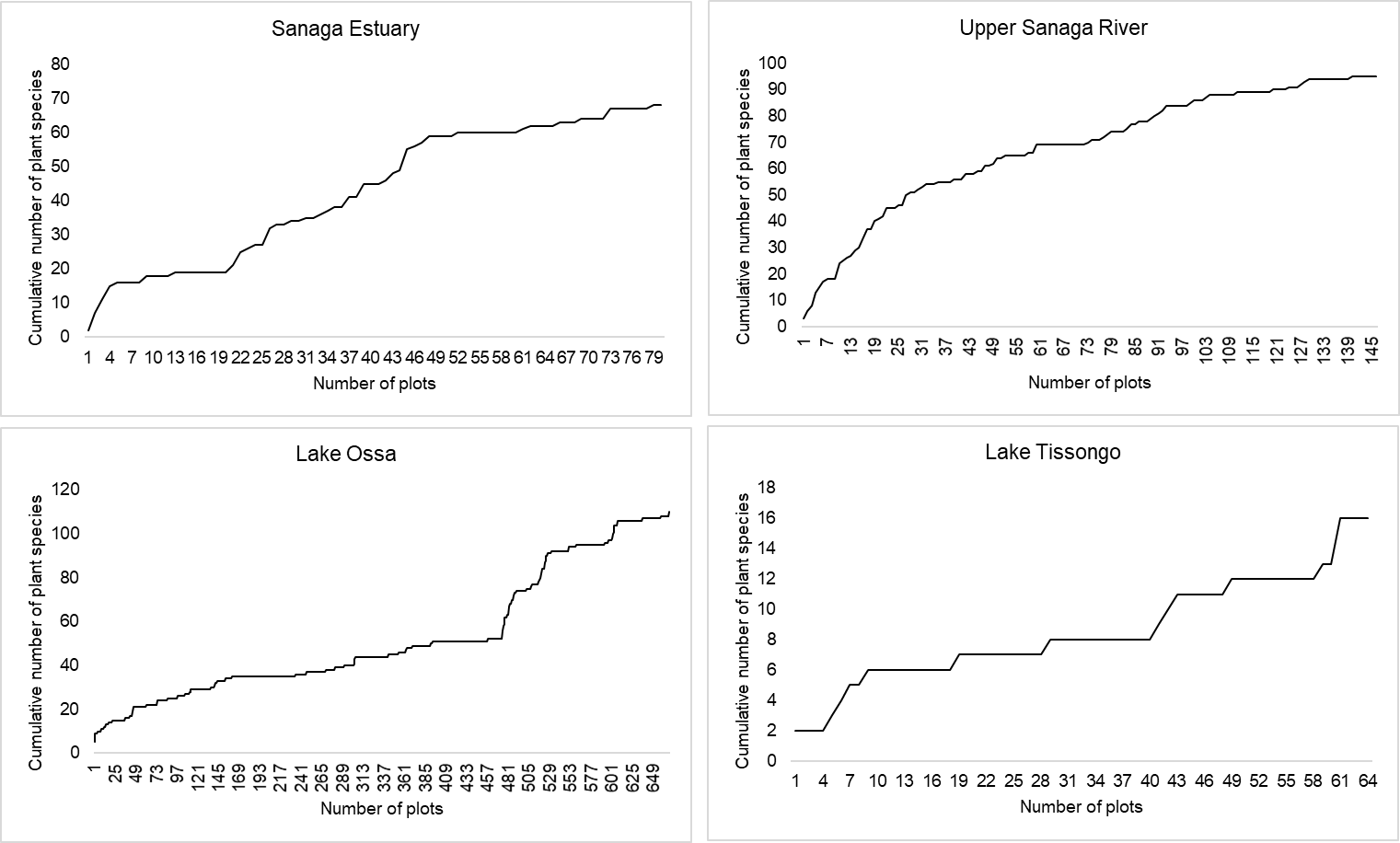


Figure S2. Cumulative number of plant species by the number of plots surveyed for each location within the downstream Sanaga River watersh


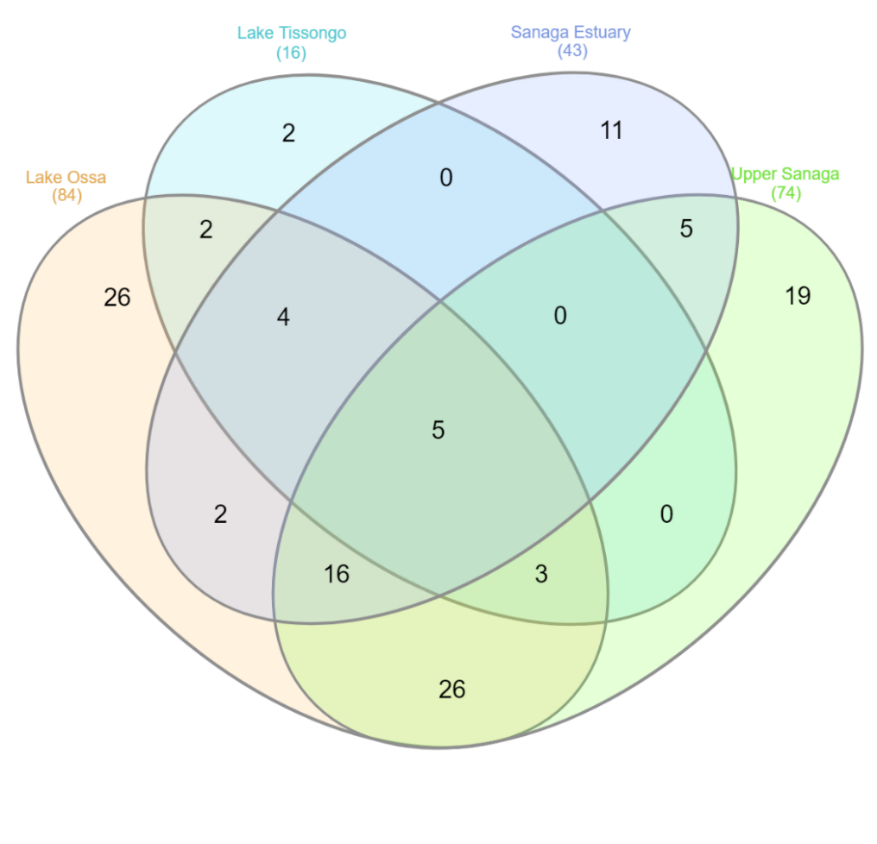


Figure S3. Venn diagram of the surveyed plant species in the four locations of the downstream Sanaga River watershed and showing shared and private species each location and combination of locations.

Figure S4. African manatee identified plant diet composition profile by season in Lake Ossa.
